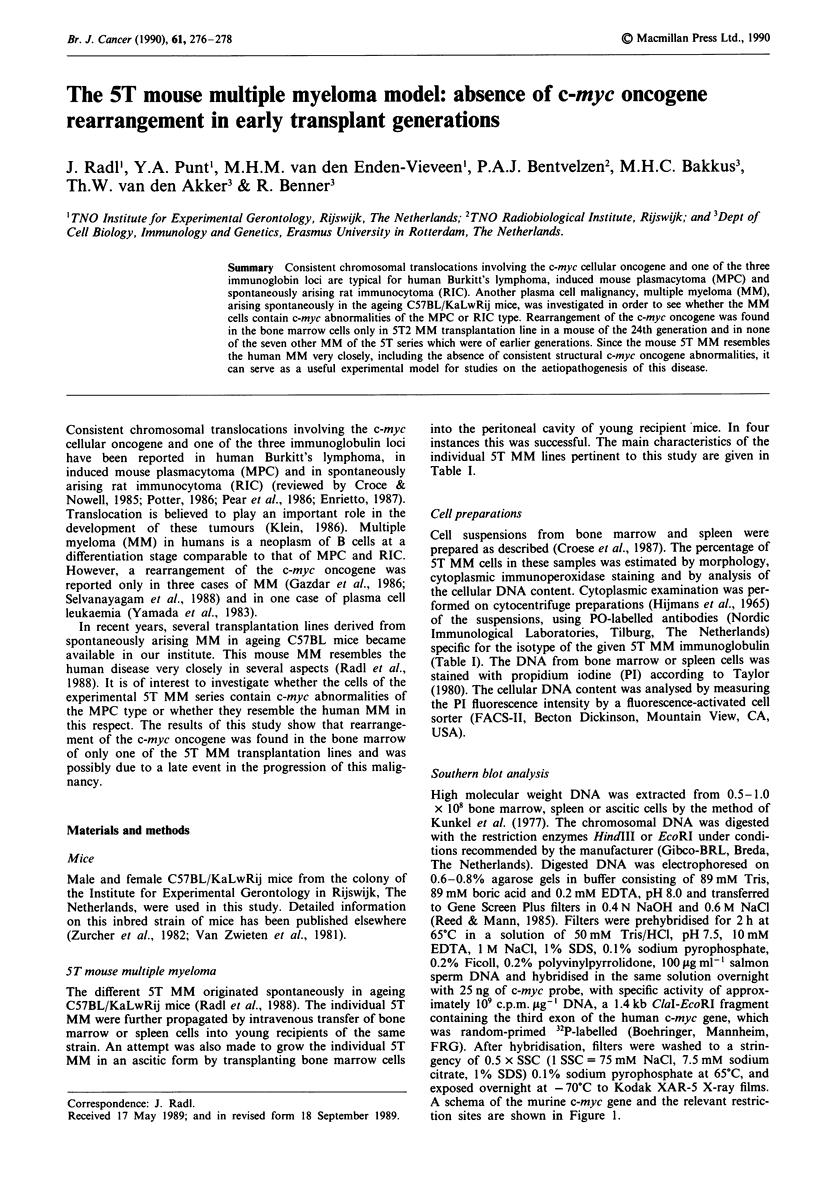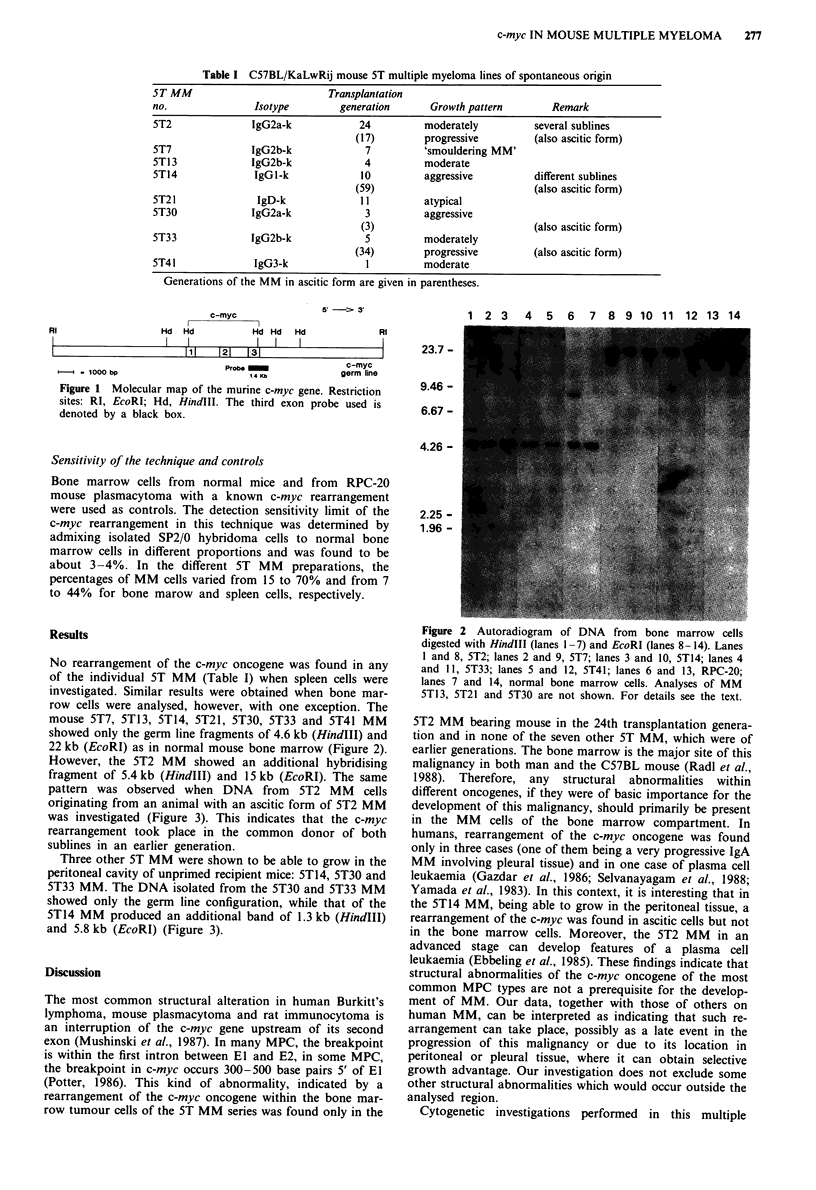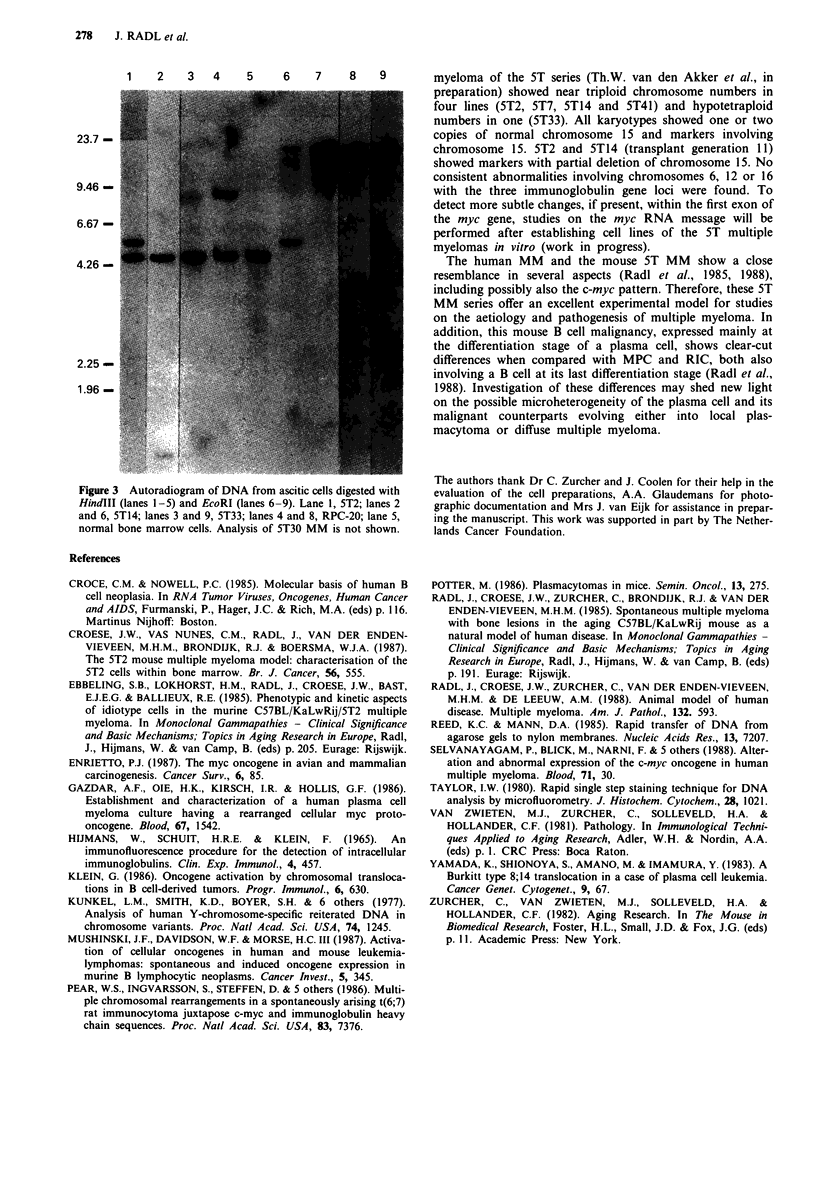# The 5T mouse multiple myeloma model: absence of c-myc oncogene rearrangement in early transplant generations.

**DOI:** 10.1038/bjc.1990.51

**Published:** 1990-02

**Authors:** J. Radl, Y. A. Punt, M. H. van den Enden-Vieveen, P. A. Bentvelzen, M. H. Bakkus, T. W. van den Akker, R. Benner

**Affiliations:** TNO Institute for Experimental Gerontology, Rijswijk, The Netherlands.

## Abstract

**Images:**


Br. J. Cancer (1990), 61, 276-278 © Macmillan Press Ltd., 1990

### The 5T mouse multiple myeloma model: absence of c-*myc* oncogene rearrangement in early transplant generations

J. Radl[1], Y.A. Punt[1], M.H.M. van den Enden-Vieveen[1], P.A.J. Bentvelzen[2], M.H.C. Bakkus[3], Th.W. van den Akker[3] & R. Benner[3]

[1]*TNO Institute for Experimental Gerontology, Rijswijk, The Netherlands;* [2]*TNO Radiobiological Institute, Rijswijk; and* [3]*Dept of Cell Biology, Immunology and Genetics, Erasmus University in Rotterdam, The Netherlands.*

**Summary** Consistent chromosomal translocations involving the c-*myc* cellular oncogene and one of the three immunoglobin loci are typical for human Burkitt's lymphoma, induced mouse plasmacytoma (MPC) and spontaneously arising rat immunocytoma (RIC). Another plasma cell malignancy, multiple myeloma (MM), arising spontaneously in the ageing C57BL/KaLwRij mice, was investigated in order to see whether the MM cells contain c-*myc* abnormalities of the MPC or RIC type. Rearrangement of the c-*myc* oncogene was found in the bone marrow cells only in 5T2 MM transplantation line in a mouse of the 24th generation and in none of the seven other MM of the 5T series which were of earlier generations. Since the mouse 5T MM resembles the human MM very closely, including the absence of consistent structural c-*myc* oncogene abnormalities, it can serve as a useful experimental model for studies on the aetiopathogenesis of this disease.

Consistent chromosomal translocations involving the c-*myc* cellular oncogene and one of the three immunoglobulin loci have been reported in human Burkitt's lymphoma, in induced mouse plasmacytoma (MPC) and in spontaneously arising rat immunocytoma (RIC) (reviewed by Croce & Nowell, 1985; Potter, 1986; Pear *et al.*, 1986; Enrietto, 1987). Translocation is believed to play an important role in the development of these tumours (Klein, 1986). Multiple myeloma (MM) in humans is a neoplasm of B cells at a differentiation stage comparable to that of MPC and RIC. However, a rearrangement of the c-*myc* oncogene was reported only in three cases of MM (Gazdar *et al.*, 1986; Selvanayagam *et al.*, 1988) and in one case of plasma cell leukaemia (Yamada *et al.*, 1983).

In recent years, several transplantation lines derived from spontaneously arising MM in ageing C57BL mice became available in our institute. This mouse MM resembles the human disease very closely in several aspects (Radl *et al.*, 1988). It is of interest to investigate whether the cells of the experimental 5T MM series contain c-*myc* abnormalities of the MPC type or whether they resemble the human MM in this respect. The results of this study show that rearrangement of the c-*myc* oncogene was found in the bone marrow of only one of the 5T MM transplantation lines and was possibly due to a late event in the progression of this malignancy.

### Materials and methods

### *Mice*

Male and female C57BL/KaLwRij mice from the colony of the Institute for Experimental Gerontology in Rijswijk, The Netherlands, were used in this study. Detailed information on this inbred strain of mice has been published elsewhere (Zurcher *et al.*, 1982; Van Zwieten *et al.*, 1981).

### *5T mouse multiple myeloma*

The different 5T MM originated spontaneously in ageing C57BL/KaLwRij mice (Radl *et al.*, 1988). The individual 5T MM were further propagated by intravenous transfer of bone marrow or spleen cells into young recipients of the same strain. An attempt was also made to grow the individual 5T MM in an ascitic form by transplanting bone marrow cells into the peritoneal cavity of young recipient mice. In four instances this was successful. The main characteristics of the individual 5T MM lines pertinent to this study are given in Table I.

### *Cell preparations*

Cell suspensions from bone marrow and spleen were prepared as described (Croese *et al.*, 1987). The percentage of 5T MM cells in these samples was estimated by morphology, cytoplasmic immunoperoxidase staining and by analysis of the cellular DNA content. Cytoplasmic examination was performed on cytocentrifuge preparations (Hijmans *et al.*, 1965) of the suspensions, using PO-labelled antibodies (Nordic Immunological Laboratories, Tilburg, The Netherlands) specific for the isotype of the given 5T MM immunoglobulin (Table I). The DNA from bone marrow or spleen cells was stained with propidium iodine (PI) according to Taylor (1980). The cellular DNA content was analysed by measuring the PI fluorescence intensity by a fluorescence-activated cell sorter (FACS-II, Becton Dickinson, Mountain View, CA, USA).

### *Southern blot analysis*

High molecular weight DNA was extracted from 0.5–1.0 $\times 10^8$ bone marrow, spleen or ascitic cells by the method of Kunkel *et al.* (1977). The chromosomal DNA was digested with the restriction enzymes *Hind*III or *Eco*RI under conditions recommended by the manufacturer (Gibco-BRL, Breda, The Netherlands). Digested DNA was electrophoresed on 0.6–0.8% agarose gels in buffer consisting of 89 mM Tris, 89 mM boric acid and 0.2 mM EDTA, pH 8.0 and transferred to Gene Screen Plus filters in 0.4 N NaOH and 0.6 M NaCl (Reed & Mann, 1985). Filters were prehybridised for 2 h at 65°C in a solution of 50 mM Tris/HCl, pH 7.5, 10 mM EDTA, 1 M NaCl, 1% SDS, 0.1% sodium pyrophosphate, 0.2% Ficoll, 0.2% polyvinylpyrrolidone, 100 $\mu g\ ml^{-1}$ salmon sperm DNA and hybridised in the same solution overnight with 25 ng of c-*myc* probe, with specific activity of approximately $10^9$ c.p.m. $\mu g^{-1}$ DNA, a 1.4 kb *Cla*I-*Eco*RI fragment containing the third exon of the human c-*myc* gene, which was random-primed $^{32}P$-labelled (Boehringer, Mannheim, FRG). After hybridisation, filters were washed to a stringency of 0.5 × SSC (1 SSC = 75 mM NaCl, 7.5 mM sodium citrate, 1% SDS) 0.1% sodium pyrophosphate at 65°C, and exposed overnight at −70°C to Kodak XAR-5 X-ray films. A schema of the murine c-*myc* gene and the relevant restriction sites are shown in Figure 1.

Correspondence: J. Radl.
Received 17 May 1989; and in revised form 18 September 1989.

**Table I** C57BL/KaLwRij mouse 5T multiple myeloma lines of spontaneous origin

| *5T MM no.* | *Isotype* | *Transplantation generation* | *Growth pattern* | *Remark* |
|---|---|---|---|---|
| 5T2 | IgG2a-k | 24 | moderately | several sublines |
| | | (17) | progressive | (also ascitic form) |
| 5T7 | IgG2b-k | 7 | 'smouldering MM' | |
| 5T13 | IgG2b-k | 4 | moderate | |
| 5T14 | IgG1-k | 10 | aggressive | different sublines |
| | | (59) | | (also ascitic form) |
| 5T21 | IgD-k | 11 | atypical | |
| 5T30 | IgG2a-k | 3 | aggressive | |
| | | (3) | | (also ascitic form) |
| 5T33 | IgG2b-k | 5 | moderately | |
| | | (34) | progressive | (also ascitic form) |
| 5T41 | IgG3-k | 1 | moderate | |

Generations of the MM in ascitic form are given in parentheses.

**Figure 1** Molecular map of the murine c-*myc* gene. Restriction sites: RI, *Eco*RI; Hd, *Hin*dIII. The third exon probe used is denoted by a black box.

*Sensitivity of the technique and controls*

Bone marrow cells from normal mice and from RPC-20 mouse plasmacytoma with a known c-*myc* rearrangement were used as controls. The detection sensitivity limit of the c-*myc* rearrangement in this technique was determined by admixing isolated SP2/0 hybridoma cells to normal bone marrow cells in different proportions and was found to be about 3–4%. In the different 5T MM preparations, the percentages of MM cells varied from 15 to 70% and from 7 to 44% for bone marow and spleen cells, respectively.

### Results

No rearrangement of the c-*myc* oncogene was found in any of the individual 5T MM (Table I) when spleen cells were investigated. Similar results were obtained when bone marrow cells were analysed, however, with one exception. The mouse 5T7, 5T13, 5T14, 5T21, 5T30, 5T33 and 5T41 MM showed only the germ line fragments of 4.6 kb (*Hin*dIII) and 22 kb (*Eco*RI) as in normal mouse bone marrow (Figure 2). However, the 5T2 MM showed an additional hybridising fragment of 5.4 kb (*Hin*dIII) and 15 kb (*Eco*RI). The same pattern was observed when DNA from 5T2 MM cells originating from an animal with an ascitic form of 5T2 MM was investigated (Figure 3). This indicates that the c-*myc* rearrangement took place in the common donor of both sublines in an earlier generation.

Three other 5T MM were shown to be able to grow in the peritoneal cavity of unprimed recipient mice: 5T14, 5T30 and 5T33 MM. The DNA isolated from the 5T30 and 5T33 MM showed only the germ line configuration, while that of the 5T14 MM produced an additional band of 1.3 kb (*Hin*dIII) and 5.8 kb (*Eco*RI) (Figure 3).

### Discussion

The most common structural alteration in human Burkitt's lymphoma, mouse plasmacytoma and rat immunocytoma is an interruption of the c-*myc* gene upstream of its second exon (Mushinski *et al.*, 1987). In many MPC, the breakpoint is within the first intron between E1 and E2, in some MPC, the breakpoint in c-*myc* occurs 300–500 base pairs 5′ of E1 (Potter, 1986). This kind of abnormality, indicated by a rearrangement of the c-*myc* oncogene within the bone marrow tumour cells of the 5T MM series was found only in the

**Figure 2** Autoradiogram of DNA from bone marrow cells digested with *Hin*dIII (lanes 1–7) and *Eco*RI (lanes 8–14). Lanes 1 and 8, 5T2; lanes 2 and 9, 5T7; lanes 3 and 10, 5T14; lanes 4 and 11, 5T33; lanes 5 and 12, 5T41; lanes 6 and 13, RPC-20; lanes 7 and 14, normal bone marrow cells. Analyses of MM 5T13, 5T21 and 5T30 are not shown. For details see the text.

5T2 MM bearing mouse in the 24th transplantation generation and in none of the seven other 5T MM, which were of earlier generations. The bone marrow is the major site of this malignancy in both man and the C57BL mouse (Radl *et al.*, 1988). Therefore, any structural abnormalities within different oncogenes, if they were of basic importance for the development of this malignancy, should primarily be present in the MM cells of the bone marrow compartment. In humans, rearrangement of the c-*myc* oncogene was found only in three cases (one of them being a very progressive IgA MM involving pleural tissue) and in one case of plasma cell leukaemia (Gazdar *et al.*, 1986; Selvanayagam *et al.*, 1988; Yamada *et al.*, 1983). In this context, it is interesting that in the 5T14 MM, being able to grow in the peritoneal tissue, a rearrangement of the c-*myc* was found in ascitic cells but not in the bone marrow cells. Moreover, the 5T2 MM in an advanced stage can develop features of a plasma cell leukaemia (Ebbeling *et al.*, 1985). These findings indicate that structural abnormalities of the c-*myc* oncogene of the most common MPC types are not a prerequisite for the development of MM. Our data, together with those of others on human MM, can be interpreted as indicating that such rearrangement can take place, possibly as a late event in the progression of this malignancy or due to its location in peritoneal or pleural tissue, where it can obtain selective growth advantage. Our investigation does not exclude some other structural abnormalities which would occur outside the analysed region.

Cytogenetic investigations performed in this multiple

**Figure 3** Autoradiogram of DNA from ascitic cells digested with *Hind*III (lanes 1–5) and *Eco*RI (lanes 6–9). Lane 1, 5T2; lanes 2 and 6, 5T14; lanes 3 and 9, 5T33; lanes 4 and 8, RPC-20; lane 5, normal bone marrow cells. Analysis of 5T30 MM is not shown.

myeloma of the 5T series (Th.W. van den Akker *et al.*, in preparation) showed near triploid chromosome numbers in four lines (5T2, 5T7, 5T14 and 5T41) and hypotetraploid numbers in one (5T33). All karyotypes showed one or two copies of normal chromosome 15 and markers involving chromosome 15. 5T2 and 5T14 (transplant generation 11) showed markers with partial deletion of chromosome 15. No consistent abnormalities involving chromosomes 6, 12 or 16 with the three immunoglobulin gene loci were found. To detect more subtle changes, if present, within the first exon of the *myc* gene, studies on the *myc* RNA message will be performed after establishing cell lines of the 5T multiple myelomas *in vitro* (work in progress).

The human MM and the mouse 5T MM show a close resemblance in several aspects (Radl *et al.*, 1985, 1988), including possibly also the c-*myc* pattern. Therefore, these 5T MM series offer an excellent experimental model for studies on the aetiology and pathogenesis of multiple myeloma. In addition, this mouse B cell malignancy, expressed mainly at the differentiation stage of a plasma cell, shows clear-cut differences when compared with MPC and RIC, both also involving a B cell at its last differentiation stage (Radl *et al.*, 1988). Investigation of these differences may shed new light on the possible microheterogeneity of the plasma cell and its malignant counterparts evolving either into local plasmacytoma or diffuse multiple myeloma.

The authors thank Dr C. Zurcher and J. Coolen for their help in the evaluation of the cell preparations, A.A. Glaudemans for photographic documentation and Mrs J. van Eijk for assistance in preparing the manuscript. This work was supported in part by The Netherlands Cancer Foundation.

### References

CROCE, C.M. & NOWELL, P.C. (1985). Molecular basis of human B cell neoplasia. In *RNA Tumor Viruses, Oncogenes, Human Cancer and AIDS*, Furmanski, P., Hager, J.C. & Rich, M.A. (eds) p. 116. Martinus Nijhoff: Boston.

CROESE, J.W., VAS NUNES, C.M., RADL, J., VAN DER ENDEN-VIEVEEN, M.H.M., BRONDIJK, R.J. & BOERSMA, W.J.A. (1987). The 5T2 mouse multiple myeloma model: characterisation of the 5T2 cells within bone marrow. *Br. J. Cancer*, **56**, 555.

EBBELING, S.B., LOKHORST, H.M., RADL, J., CROESE, J.W., BAST, E.J.E.G. & BALLIEUX, R.E. (1985). Phenotypic and kinetic aspects of idiotype cells in the murine C57BL/KaLwRij/5T2 multiple myeloma. In *Monoclonal Gammapathies – Clinical Significance and Basic Mechanisms; Topics in Aging Research in Europe*, Radl, J., Hijmans, W. & van Camp, B. (eds) p. 205. Eurage: Rijswijk.

ENRIETTO, P.J. (1987). The myc oncogene in avian and mammalian carcinogenesis. *Cancer Surv.*, **6**, 85.

GAZDAR, A.F., OIE, H.K., KIRSCH, I.R. & HOLLIS, G.F. (1986). Establishment and characterization of a human plasma cell myeloma culture having a rearranged cellular myc proto-oncogene. *Blood*, **67**, 1542.

HIJMANS, W., SCHUIT, H.R.E. & KLEIN, F. (1965). An immunofluorescence procedure for the detection of intracellular immunoglobulins. *Clin. Exp. Immunol.*, **4**, 457.

KLEIN, G. (1986). Oncogene activation by chromosomal translocations in B cell-derived tumors. *Progr. Immunol.*, **6**, 630.

KUNKEL, L.M., SMITH, K.D., BOYER, S.H. & 6 others (1977). Analysis of human Y-chromosome-specific reiterated DNA in chromosome variants. *Proc. Natl Acad. Sci. USA*, **74**, 1245.

MUSHINSKI, J.F., DAVIDSON, W.F. & MORSE, H.C. III (1987). Activation of cellular oncogenes in human and mouse leukemia-lymphomas: spontaneous and induced oncogene expression in murine B lymphocytic neoplasms. *Cancer Invest.*, **5**, 345.

PEAR, W.S., INGVARSSON, S., STEFFEN, D. & 5 others (1986). Multiple chromosomal rearrangements in a spontaneously arising t(6;7) rat immunocytoma juxtapose c-myc and immunoglobulin heavy chain sequences. *Proc. Natl Acad. Sci. USA*, **83**, 7376.

POTTER, M. (1986). Plasmacytomas in mice. *Semin. Oncol.*, **13**, 275.

RADL, J., CROESE, J.W., ZURCHER, C., BRONDIJK, R.J. & VAN DER ENDEN-VIEVEEN, M.H.M. (1985). Spontaneous multiple myeloma with bone lesions in the aging C57BL/KaLwRij mouse as a natural model of human disease. In *Monoclonal Gammapathies – Clinical Significance and Basic Mechanisms; Topics in Aging Research in Europe*, Radl, J., Hijmans, W. & van Camp, B. (eds) p. 191. Eurage: Rijswijk.

RADL, J., CROESE, J.W., ZURCHER, C., VAN DER ENDEN-VIEVEEN, M.H.M. & DE LEEUW, A.M. (1988). Animal model of human disease. Multiple myeloma. *Am. J. Pathol.*, **132**. 593.

REED, K.C. & MANN, D.A. (1985). Rapid transfer of DNA from agarose gels to nylon membranes. *Nucleic Acids Res.*, **13**, 7207.

SELVANAYAGAM, P., BLICK, M., NARNI, F. & 5 others (1988). Alteration and abnormal expression of the c-*myc* oncogene in human multiple myeloma. *Blood*, **71**, 30.

TAYLOR, I.W. (1980). Rapid single step staining technique for DNA analysis by microfluorometry. *J. Histochem. Cytochem.*, **28**, 1021.

VAN ZWIETEN, M.J., ZURCHER, C., SOLLEVELD, H.A. & HOLLANDER, C.F. (1981). Pathology. In *Immunological Techniques Applied to Aging Research*, Adler, W.H. & Nordin, A.A. (eds) p. 1. CRC Press: Boca Raton.

YAMADA, K., SHIONOYA, S., AMANO, M. & IMAMURA, Y. (1983). A Burkitt type 8;14 translocation in a case of plasma cell leukemia. *Cancer Genet. Cytogenet.*, **9**, 67.

ZURCHER, C., VAN ZWIETEN, M.J., SOLLEVELD, H.A. & HOLLANDER, C.F. (1982). Aging Research. In *The Mouse in Biomedical Research*, Foster, H.L., Small, J.D. & Fox, J.G. (eds) p. 11. Academic Press: New York.